# Characterization of Chromosomal Structural Polymorphism in *Roegneria nutans* from the Western Sichuan Plateau, China Based on Tandem Repeat Sequences

**DOI:** 10.3390/plants15182832

**Published:** 2026-09-16

**Authors:** Cairong Yang, Yueju Zhou, Yi Ou, Zhimeng Wang, Yutong Qiao, Dingfang Luo, Jiaping Zhou, Xin Lin, Jiezhi Yang

**Affiliations:** 1 College of Chemistry and Life Science, Chengdu Normal University, Chengdu 611130, China; cairongyang828@hotmail.com (C.Y.);; 2 Sichuan Provincial Forestry and Grassland Key Laboratory of Biodiversity Conservation and Sustainable Community Development in Giant Panda National Park, Chengdu 611130, China; 3 Neijiang Academy of Agricultural Sciences, Neijiang 641000, China

**Keywords:** genome, polymorphism, genetic diversity, elevation, adaptability

## Abstract

*Roegneria nutans*, a premium grass on Western Sichuan Plateau, China, is vital for ecological stability and pastoral security. Studying its chromosomal architecture and environmental correlations lays a foundation for *Roegneria* genetic evolution research. This study used genomic in situ hybridization (GISH) and non-denaturing fluorescence in situ hybridization (ND-FISH) with two tandem repetitive sequence probes pAs1 and (AAG)_10_) to analyze the species’ St and Y genomes of *R. nutans* from different *altitudes*. Our findings demonstrated that for both the number of probe signals and variant types, inter-population variation was higher than intra-population variation. Distinct altitudinal response patterns of (AAG)_10_ and pAs1 FISH signals were detected between the St and Y genomes. Within the St genome, the abundance of (AAG)_10_ loci exhibited a trend of initial decline followed by a marked rise with increasing altitude. The pAs1 probe displayed higher signal density than (AAG)_10_, with its signal abundance peaking in medium-altitude populations, and polymorphic loci were most abundant in high-altitude populations. In contrast, medium-altitude samples harboured relatively fewer (AAG)_10_ signals within the Y genome. Although pAs1 exhibited lower signal density than (AAG)_10_ in the Y genome, its maximum signal number was observed in medium-altitude populations, where polymorphic loci predominated. Low-altitude populations maintained relatively conserved hybridization patterns, and chromosomal variation was mainly detected in the Y genome at medium altitudes and in the St genome at high altitudes. This study provides a valuable basis for exploring the genetic diversity of *R. nutans* across the Western Sichuan Plateau.

## 1. Introduction

Biodiversity typically encompasses genetic diversity, species diversity, and ecosystem diversity. As a product of Earth’s life evolving over billions of years, biodiversity is an important manifestation of the evolutionary process of life [1]. There are 35 biodiversity hotspots worldwide, covering less than 20% of Earth’s land area but harboring over half of the global plant species and nearly 80% of terrestrial vertebrates, exhibiting extremely rich biodiversity [2]. The Tibet–Himalaya–Hengduan Mountains, a unique biodiversity region, have been profoundly shaped by the synergistic effects of geology and climate, which have provided distinctive conditions for the evolution of alpine plants in the area. Ancestral groups of extant alpine plants achieved significant diversification from the early to middle Miocene [3]. This process not only forged the unique biological communities of the region but also made it one of the most valuable areas for global biodiversity research. The Western Sichuan Plateau, located in the southeastern part of the Qinghai–Tibet Plateau, is one of the world’s biodiversity conservation hotspots. Characterized by high altitude, hypoxia, and strong radiation, it is the largest alpine pasture in Sichuan and an important ecological security barrier in western China [4]. The abundant biological resources in the region provide excellent materials for biodiversity-related research.

The genus *Roegneria* C. Koch was established by Koch in 1848, with *Roegneria caucasica* C. as the type species [5]. Approximately 130 species of this genus are distributed worldwide, mainly in temperate regions of the Northern Hemisphere, Central Asia, and the Himalayas. China is home to about 70 species, primarily in Southwest, Northwest, and North China, with only one species found in Southern Europe, spanning an altitude range of 300 to 5700 m [6]. *Roegneria* includes many excellent perennial forage grasses, such as *Roegneria nutans* and *Roegneria ciliaris*. In the tribe Triticeae, species containing the St and Y genomes are classified into *Roegneria*, all of which are perennial plants. Most of them are tetraploids (2n = 4x = 28) with the genome composition StY, and some are hexaploids (2n = 6x = 42) with the genome composition StStY [6,7]. The St genome of *Roegneria* was derived from the genus *Pseudoroegneria* (Neveski) Löve, while the donor of the Y genome remains unclear [8,9]. Some scholars speculated that the diploid ancestor of the Y genome may have originated in Central Asia or the Himalayas and may be extinct [10]. Single-copy nuclear gene data strongly supported the independent origin of the Y genome [11,12], while other studies suggested that the Y and St genomes share certain homology or that the Y genome originated from the St genome [13,14].

Plant polyploidy refers to the presence of three or more sets of chromosomes in somatic cells. Polyploidy is very common in angiosperms, accounting for 30% to 70% of angiosperm species [15,16]. Existing studies have shown that polyploidization events are closely associated with environmental stress, and polyploidization can enhance the ability of organisms to rapidly adapt to extreme environmental changes [17]. Due to genome doubling, polyploid plants usually have stronger colonization ability or occupy a wider ecological niche than diploids [18]. Approximately 3 to 4 million years ago, the flora of the Holarctic region began to develop. Since then, the region has experienced frequent and repeated drastic climate and environmental changes, such as alternations between glacial and interglacial periods and sea-level fluctuations. These complex environmental changes were likely to have promoted numerous polyploidization events [19]. Polyploid species are unevenly distributed on Earth, with their occurrence frequency increasing with latitude [20]. Intergenomic rearrangements in allopolyploids may also be affected by environmental factors, leading to genetic changes that increase species’ genetic diversity and environmental adaptability, enabling their survival in different natural environments. Moraes et al. [21] conducted comparative experiments on polyploid and non-polyploid orchid species and found that genome polyploidization can alter plant genetic phenotypes, promote genomic diversification, enhance plant environmental adaptability, and ultimately drive plant evolution. In the tribe Triticeae of the Poaceae family, populations in cold alpine and grassland environments have significantly higher chromosome translocation frequencies than those in river valley and lake basin populations, and the types of chromosome translocations show a positive correlation with altitude [22]. In *Elymus dahuricus*, compared with populations at 2600–3800 m above sea level, populations at high altitudes (4150 m) have more chromosomes involved in translocations, showing more complex intergenomic translocation types, which may promote the adaptation of newly formed allopolyploids to variable and harsh environments [23]. There was a significant correlation between nuclear DNA content and altitude in *Elymus nutans*, *E. burchan-buddae*, and *E. sibiricus* [24]. It is evident that harsh habitats exert strong selective pressure on plants, and the genetics of polyploid plants are influenced by environmental factors during species evolution. In-depth exploration of the processes and intrinsic mechanisms of organisms’ adaptation to extreme habitats is of crucial significance for understanding the origin and evolution of biodiversity.

Tandem repeat sequences account for a considerable proportion of eukaryotic genomes and play vital roles in gene expression regulation, maintenance of chromosome integrity, and genomic stability [25,26]. Based on length and repeat unit size, tandem repeat sequences are classified into microsatellite DNA, minisatellite DNA, and satellite DNA [27]. These sequences are localized to specific chromosomal regions, such as telomeres, centromeres, and chromosome arms. Due to their high copy number and chromosomal specificity, they are often developed into fluorescent probes for fluorescence in situ hybridization (FISH), which is widely used in the detection of exogenous genetic material in crop breeding [28]. Tandem repeat sequences are not only products of genome polyploidization but also DNA-level imprints of evolutionary events such as genomic differentiation and speciation [29], making them effective tools for studying species origin and evolution at the molecular level. Environmental stress (e.g., ultraviolet radiation, chemical stimulation) can increase the error rate during DNA replication or repair, thereby inducing slip-strand mispairing of tandem repeat sequences and ultimately leading to variations in sequence length. When tandem repeat sequences exhibit specific length variations, plants can gain survival or adaptive advantages under certain environmental conditions [30]. Analysis of the genomes of 220 green plant species, including *Arabidopsis thaliana*, revealed that the number and function of clustered tandemly duplicated genes (CTDGs) are closely associated with the adaptive evolution of land plants [31].

The interaction between genetics and the environment is the basis of plant adaptive evolution. Environmental changes drive genetic variations in plants through selective pressure, thereby enhancing their adaptability to adverse conditions. As a dominant forage species on the Western Sichuan Plateau, *R. nutans* grows in grasslands, slopes, and riverbank meadows at altitudes of 3000 to 5000 m. It possesses excellent characteristics such as strong tillering ability, tolerance to poor soil, cold resistance, and drought resistance. Variations in its tandem repeat sequences may be driven by environmental factors and contribute to enhancing its adaptability in harsh environments. In this study, genomic in situ hybridization (GISH) and non-denaturing fluorescence in situ hybridization (ND-FISH) techniques were used, with two oligonucleotide tandem repeat sequences (pAs1 and (AAG)_10_) as probes, to characterize the chromosomal structure of *R. nutans* from different altitudes and analyze its chromosomal genetic diversity. This research supplements the basis for clarifying the genetic evolution mechanism of *Roegneria* species and is of great value for enriching biodiversity research in the region.

## 2. Materials and Methods

### 2.1. Plant Materials

Three samples of *R*. Keng (2n = 4x = 28, genome composition StStYY) were all collected from Garzê Tibetan Autonomous Prefecture, Sichuan Province, China. The collection sites ranged in altitude from 2939 to 4278 m. For sampling, one individual plant was collected every 10 m, with five plants sampled at each altitude. Detailed information of the collection sites is provided in Table 1.

### 2.2. Root Tip Collection

Seeds of *R. nutans* from each individual at each altitude were placed in Petri dishes lined with 3–4 layers of moist filter paper. They were vernalized at 4 °C for 2–3 days and then germinated in an incubator at 23 °C. When the seed roots grew to a length of 1–1.5 cm, the root tips were excised.

### 2.3. Slide Preparation

The protocol was performed referring to Jiang et al. [32] with minor modifications.

### 2.4. Pretreatment and Fixation

Excised root tips were placed in 1.5 mL Eppendorf (EP) tubes filled with distilled water. The tubes were placed in a foam box with ice and pretreated in a 4 °C refrigerator for approximately 30 h. After pretreatment, the distilled water was aspirated, and an appropriate amount of 90% glacial acetic acid was added to the EP tubes using a pipette for fixation. Fixation lasted 10–15 min. After fixation, the root tips were transferred to Petri dishes containing distilled water and rinsed sequentially with forceps for subsequent use.

### 2.5. Enzymatic Digestion

Root tips were picked out, and the surface water was blotted dry with absorbent paper. The meristematic regions of the root tips were cut off with a blade and placed in 20 μL of enzyme solution containing 4% cellulase and 2% pectinase. Enzymatic digestion was carried out in a 37 °C water bath for approximately 50 min, with the duration adjusted according to the size of the root tips and room temperature.

### 2.6. Suspension Preparation

After digestion, the enzyme solution was completely aspirated. The root tips were rinsed twice with 70% ethanol, and a small amount of ethanol was retained in the EP tube after the final rinse. The root tips were mashed gently with a dissecting needle to avoid damaging the chromosome structure. The EP tubes with mashed root tips were centrifuged at 6000 r/min for 2 min. After centrifugation, the supernatant was discarded. Once the tube was air-dried naturally, an appropriate amount of pure glacial acetic acid was added and mixed thoroughly.

### 2.7. Slide Dropping and Observation

A 5 μL aliquot of the root tip suspension was pipetted onto slides for dropping. The slides were observed under a microscope, and those with well-dispersed chromosomes were stored in a dry environment for subsequent experiments.

### 2.8. Probe Preparation

Genomic DNA of *Pseudoroegneria libanotica* (2n = 2x = 14, StSt) was extracted using a Plant Genomic DNA Rapid Extraction Kit (Sangon Biotech Co., Ltd., Shanghai, China) and labeled with the Atto 550 NT (red) DNA Labeling Kit (Jena Bioscience, Jena, Germany). Oligonucleotide probes used for FISH were synthesized by Sangon Biotech Co., Ltd. The (AAG)_10_ probe was labeled with TAMRA (red) fluorescent group at the 5′ end, while pAs1 was labeled with FAM (green) fluorescent group at the 5′ end.

### 2.9. In Situ Hybridization

The experiment was performed referring to Tao et al. [33] with minor modifications.

#### 2.9.1. Non-Denaturing Fluorescence In Situ Hybridization (ND-FISH)

Preparation of hybridization chambers and chromosome-bearing slides: 3–4 layers of filter paper were placed at the bottom of hybridization chambers and moistened with distilled water (not excessive to prevent water from entering the slides). The chambers were preheated in a 42 °C incubator for 30 min.

Preparation of hybridization mixture: The hybridization mixture consisted of probes, TE buffer, and 2 × SSC buffer. It was prepared at a volume of 10 μL per slide. Typically, 1 μL of probe dilution (10 μmol/mL) was used per probe per slide, and the concentration of the probe dilution could be adjusted according to the hybridization signal intensity. The hybridization mixture was prepared on ice.

Fluorescence in situ hybridization: 10 μL of the prepared hybridization mixture was dropped onto the marked area of the slide, covered with a coverslip, and the slide was placed in the preheated hybridization chamber. Hybridization was carried out at 42 °C for 2 h.

Slide washing and staining: After hybridization, the slides were removed from the hybridization chamber and placed in 2 × SSC solution to allow the coverslips to fall off naturally. The slides were then rinsed in distilled water, dried with a rubber bulb, and 12 μL of DAPI was dropped onto the marked area. A coverslip was added, and the slides were incubated in the dark for 15 min for staining.

#### 2.9.2. Genomic In Situ Hybridization (GISH)

After ND-FISH, the slides were recovered by soaking in 70% ethanol preheated to 60 °C for 5 min, followed by GISH. The experimental procedure was the same as that of ND-FISH, except that the slides were denatured in a boiling water bath for 2 min before adding the hybridization mixture, and the hybridization was carried out overnight.

### 2.10. Microscopic Observation

Microscopic observation was performed using a Leica binocular fluorescence microscope (DM4B) equipped with a DFC7000T imaging system (Leica Microsystems GmbH, Wetzlar, Germany). Chromosomes with different fluorescent signals were observed under an oil immersion lens. Three cells were observed per individual plant at each altitude. Subsequent image analysis was completed using Adobe Photoshop (2024 version) software.

### 2.11. Statistics of Hybridization Signals

Chromosomes are structurally divided into short (S) and long (L) arms, with the centromere acting as the fundamental positional landmark. According to the physical localization of fluorescent hybridization signals, each chromosome can be partitioned into four functional chromosomal domains: the centromeric, pericentromeric, subtelomeric, and telomeric regions. A valid hybridization locus is defined as a discrete punctate or diffuse fluorescent signal on the chromosome whose intensity is markedly higher than the background fluorescence level. Signals distributed at distinct positions on the same chromosomal arm are recorded as individual loci. Weak fluorescent signals, diffuse background noise, and non-specific signals are excluded from subsequent statistical analysis.

For homologous chromosome pairs, a hybridization locus is categorized as polymorphic if it satisfies any of the following four criteria. First, presence–absence polymorphism, in which a specific chromosomal region exhibits a detectable hybridization signal on one homologous chromosome but no corresponding signal on its counterpart. Second, positional polymorphism, where both homologous chromosomes generate valid hybridization signals, whereas the physical locations of these signals differ. Third, copy-number polymorphism, referring to significant variation in signal counts at identical chromosomal loci between homologous chromosomes; for example, one homolog displays a single signal site while the other presents two or more signal sites. Fourth, intensity polymorphism, characterized by prominent differences in fluorescent signal brightness, which is verified by two independent evaluators. In contrast, loci with stable signal existence, consistent positional distribution and uniform intensity across all tested samples are defined as conserved loci and excluded from polymorphism statistical assessment.

The mean ± standard deviation (SD) of probe signals was calculated in Microsoft Excel 2021. One-way analysis of variance (ANOVA) was conducted, and significant differences among groups were evaluated by multiple comparison tests with SPSS 26.0.

## 3. Results

### 3.1. Identification of Genomes and Individual Chromosomes in R. nutans

**Figure 1:**
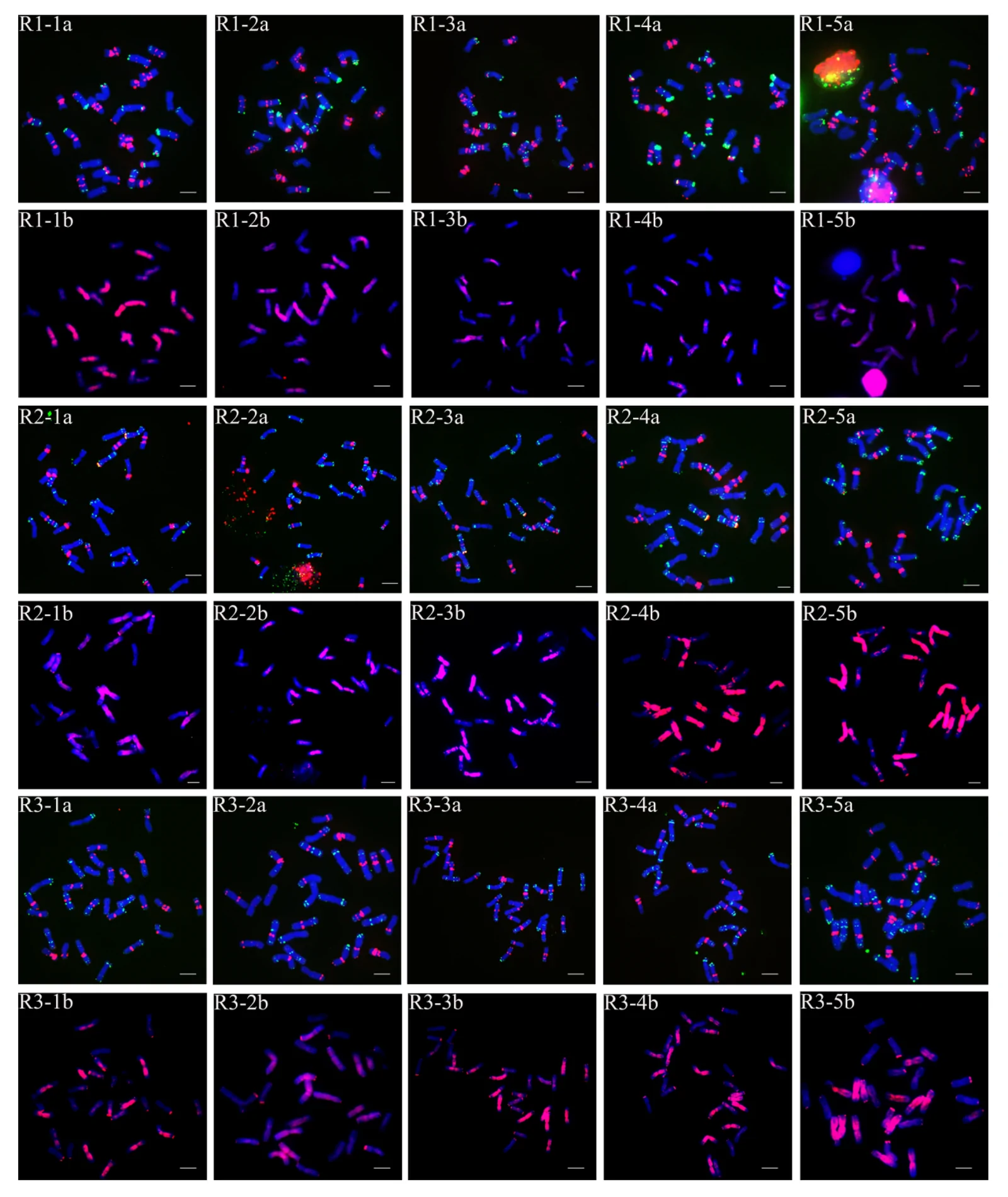
Distribution patterns of (AAG)_10_, and pAs1 repetitive sequence probe combinations on chromosomes of *Roegneria nutans* from three different altitudes. Annotation: R1, R2 and R3 represent R. nutans accessions RNU0403, RNU0408, and RNU0416, respectively. The numbers following R1, R2 and R3 denote five individual plants from the corresponding populations. R1-a, R2-a and R3-a: FISH signal patterns of (AAG)_10_ (red) and pAs1 (green) probes. R1-b, R2-b and R3-b: GISH signals using *Pseudoroegneria libanotica* genomic DNA as the probe (red). Scale bar = 10 μm.

**Figure 2:**
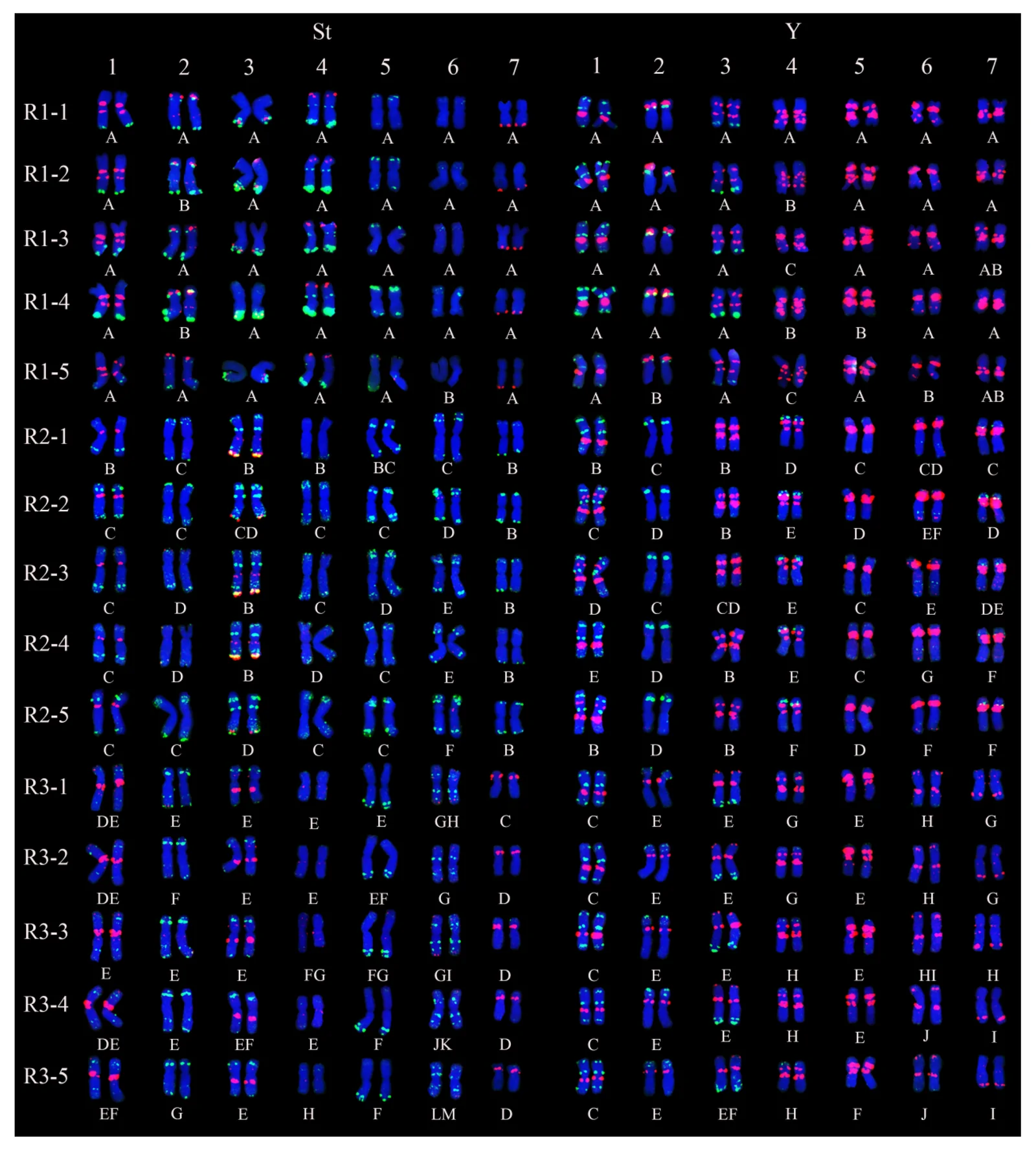
Molecular karyotype analysis of three *R. nutans* samples from different altitudes based on the (AAG)_10_ (red) and pAs1 (green) probe combination. Annotation: Taking the signal pattern of each numbered chromosome of individual R1-1 as the reference type, it is denoted as type A. For the chromosomes of other materials under the same genome and the same number, conduct comparison and classification according to the signal patterns: those with signal patterns consistent with the reference type are classified into the same type; when a new signal pattern different from the existing types appears, it is named sequentially as in alphabetical order, which is the variant type of that chromosome number.

Three *R. nutans* samples were collected from different altitudes. After sequential FISH-GISH, it was consistently observed that all samples had 28 chromosomes. Due to the partial homology between the St and Y genomes, the signals from GISH allowed the chromosomes of *R. nutans* to be roughly divided into two genomes: St (red) and Y (blue). Chromosomes within each genome were numbered 1–7 based on parameters including probe signal characteristics, relative length, and the ratio of short arm to long arm length (Figure 1 and Figure 2).

### 3.2. Distribution of Tandem Repeat Sequences in the St Genome of R. nutans

FISH results from the combination of (AAG)_10_, and pAs1 probes are shown in Figure 1 and Figure 2. Signals of (AAG)_10_ were primarily concentrated in the subtelomeric, pericentromeric, and centromeric regions, mostly with a punctate distribution. Within populations, except for signals generated by the (AAG)_10_ probe on the St genome in the mid-altitude population R2, which exhibited moderate variation among individual plants, signal polymorphisms among individuals were relatively low in the remaining populations (low-altitude population R1 and high-altitude population R3). The average number of (AAG)_10_ loci was 8.0, 5.2, and 16.2 in low-, medium-, and high-altitude samples, respectively, showing an initial decrease followed by a continuous increase with rising altitude (Table 2 and Table S1). The number of signals in the high-altitude population was significantly higher than those in the middle- and low-altitude populations. The hybridization signals presented a punctate and invariable pattern in the low-altitude population, whereas signal loci exhibited a certain degree of variation in the middle- and high-altitude populations. No polymorphic loci were detected in materials from low and middle altitudes, whereas a total of 10 polymorphic loci were identified in high-altitude material. Among high-altitude individuals, chromosome 6St exhibited the maximum number of polymorphic loci. Hybridization signals of pAs1 were mainly distributed in the telomeric, subtelomeric, centromeric, and pericentromeric regions of St genome chromosomes, mostly in a punctate pattern. The average hybridization locus number displayed a rise and subsequent fall across altitudinal groups, with values of 12.2, 28.6, and 20 at low, middle, and high altitudes, respectively. Significant differences were observed in the number of signals among different populations. There was also a certain degree of variation in signal number among individual plants within each population. The numbers of polymorphic loci were 1, 2, and 3 in low-, middle-, and high-altitude materials, respectively, showing a gradual increase with rising altitude. Overall, chromosomes 1 St and 5St exhibited relatively high polymorphism. In terms of both signal abundance and polymorphic loci, the pAs1 probe displayed high density with low variability on St genome among different populations. Overall, within the St genome, the number of (AAG)_10_ signals increase with rising altitude, pAs1 exhibits a higher signal density than (AAG)_10_ with its signal peak occurring in the medium-altitude population, and polymorphic loci are most abundant in high-altitude populations.

### 3.3. Distribution of Tandem Repeat Sequences in the Y Genome of R. nutans

The (AAG)_10_ hybridization signals mainly accumulated in the telomeric, subtelomeric, pericentromeric, and centromeric chromosomal regions and predominantly exhibited a punctate distribution pattern (Figure 1 and Figure 2). Signal numbers exhibited limited variation among individual plants within all populations. The average numbers of hybridization loci were 18.2, 16.8, and 18.2 in low-, middle-, and high-altitude materials, respectively (Table 2 and Table S1). The number of signal loci in the middle-altitude population was significantly lower than that in the low- and high-altitude populations. Nevertheless, intra-population variation in signal counts was comparatively greater in the middle-altitude population, while it was relatively stable in low- and high-altitude populations. Materials from low and middle altitudes both possessed four polymorphic loci, while high-altitude materials contained only two polymorphic loci. The chromosomes harboring polymorphic loci differed among materials from different altitudes. The distribution of pAs1 hybridization signals in the Y genome was consistent with that of (AAG)_10_, mainly localized in the telomeric, subtelomeric, and centromeric regions. Low-altitude, middle-altitude, and high-altitude materials exhibited average hybridization locus numbers of 6.6, 16.8, and 10.8, respectively, indicating an upward trend from low to middle altitude and a subsequent drop at high altitude. Significant differences in signal number were detected among populations across altitudinal gradients. Consistent with the signal pattern of the (AAG)_10_ probe, the number of pAs1 signals also exhibited relatively large variation among individual plants within the middle-altitude population, whereas it remained relatively stable in populations originating from low and high altitudes. No polymorphic loci were detected in low-altitude materials, while two and one polymorphic loci were observed in middle- and high-altitude materials, respectively. Additionally, chromosome 3St harbored one polymorphic locus in both middle- and high-altitude populations. Compared with low-altitude populations, the pAs1 probe exhibited high density and low variability on the Y genome in middle- and high-altitude populations, especially in middle-altitude population. Overall, within the Y genome, the number of (AAG)_10_ signals is relatively low in the medium-altitude population, pAs1 displays a lower signal density than (AAG)_10_ with its signal-number peak occurring in the medium-altitude population, and polymorphic loci are more numerous in the medium-altitude population.

### 3.4. Chromosomal Polymorphism of Different Samples Characterized by Combinations of Tandem Repeat Probes

Variation types within and among populations were identified and quantified based on the signal patterns of (AAG)_10_ + pAs1 probes (Figure 2, Table 3). Results showed that inter-population variation exceeded intra-population variation across all populations. St genome had more variation types (51) than Y genome (49) (Table 3). Chromosome 6St and 6Y exhibited the highest variation, with 13 and 10 variation types, respectively. At the intrapopulation level, signal patterns were relatively conserved in the low-altitude population. Chromosomes 1St, 3St, 4St, 5St, 7St, 1Y and 3Y shared consistent signal patterns without any variation within the population. The remaining chromosomes also exhibited limited variation types, with only two types observed. Intrapopulation variation types were more abundant in the middle-altitude population than in the low-altitude population. Chromosomes 6Y, 1Y and 7Y presented 5, 4 and 4 variation types, respectively. The number of variation types of the St genome within the middle-altitude population was also higher than that in the low-altitude population. Chromosome 6St showed extensive variation in the high-altitude population, with a total of seven variation types identified. The variation in Y-genome chromosomes in the high-altitude population was lower than that in the middle-altitude population. Overall, according to the signal distribution patterns of (AAG)_10_ + pAs1 probes, the low-altitude population possessed relatively conserved signal patterns, the middle-altitude population showed high variation in the Y genome, and the high-altitude population presented prominent variation in the St genome.

## 4. Discussion

### 4.1. Analysis of Chromosomal Structural Polymorphism in R. nutans

Using ND-FISH and GISH techniques, we successfully distinguished the two genomes (St and Y) of *R. nutans* and the seven pairs of chromosomes within each genome. The (AAG)_10_ probe also had more signals in the Y genome. In contrast, pAs1 was more enriched in the St genome. Liu et al. [34] analyzed the chromosomal polymorphism of *R. nutans* from Qinghai using FISH and found that pAs1 signals were stronger in the St genome with fewer AAG loci, while the Y genome showed weaker pAs1 signals and more AAG locus variations, which was consistent with the results of this study. Compared to *Roegneria* species containing the St genome, more studies have focused on *Elymus* species with St and Y genomes. Dou et al. [35] reported that using pAs1 and (AAG)_10_ probes, the Y genome of *Elymus nutans* from the Qinghai–Tibet Plateau displayed more chromosomal variations than the St genome, with variations mainly manifested as tandem repeat deletion, amplification, and intergenomic translocation. Previous studies on the allohexaploid *E*. *nutans* (with fertility differences) from the Qinghai–Tibet Plateau, using pSc119.2, pTa71-2, pAs1, and (AAG)_10_ probes for chromosomal structure analysis, revealed a decreasing trend of polymorphism from the Y genome to the St genome and then to the H genome [36]. These findings differ from the chromosomal polymorphism results of the present study, which revealed that the St genome harbours more polymorphic loci and greater variation types than the Y genome. However, Tao et al. [33] found that the St genome of *Kengyilia hirsuta* had more chromosomal variations than the Y genome, which is in accordance with the results of this study. This discrepancy suggested that genomic variations during polyploidization varied among Triticeae species. Based on the molecular karyotype of *R. nutans* in this study, three samples from different altitudes exhibited rich chromosomal polymorphism. This is consistent with Liu et al. [37], who used two FISH probes (S5 and (AAG)_10_) and found variations within and among four wild *E*. *nutans* populations, indicating high genetic diversity at the chromosomal level. The chromosomal polymorphism of *R. nutans* based on the pAs1 probe was mainly concentrated in the centromeric and terminal regions of chromosomes. This is in agreement with Wang et al. [38], who reported that pAs1 detected chromosomal polymorphism mainly in the terminal regions (and a few in the centromeric regions) of the St, Y, and P genomes of *Kengyilia grandiglumis*. Genetic diversity is the foundation for populations to adapt to environmental changes, and the level of genetic diversity reflects a species’ ability to adapt to the environment-especially crucial under climate change [39]. As a key forage species in the Western Sichuan Plateau ecosystem, this study revealed abundant chromosomal structural polymorphism in *R. nutans* across different altitudes, which is an evolutionary product of the species’ long-term adaptation to the unique plateau environment. Meanwhile, it plays an irreplaceable core role in maintaining the stability and functional integrity of regional ecosystems and ensuring ecological security. Strengthening the conservation of *R. nutans* genetic diversity in the future holds important theoretical and practical significance.

### 4.2. Impact of Altitude on Chromosomal Structural Polymorphism in R. nutans

During genome evolution, tandem repeat sequences exhibit characteristics of rapid generation, elimination, and variation, which are generally attributed to the frequent occurrence and gradual accumulation of base differences in their tandem repeat units [40]. Distant hybridization and polyploidization are important factors inducing variations in tandem repeat sequences such as microsatellite DNA and promoting their coevolution [41]. Microsatellite sequences in plants can participate in individual development and play a key role in species survival and evolutionary adaptation [42]. In this study, two tandem repeat probes pAs1, and (AAG)_10_) were used to analyze the chromosomal structure of *R. nutans* samples from different altitudes. The results showed that medium and high-altitude samples generally had higher chromosomal structural polymorphism than low-altitude samples. This difference may be related to adaptive evolution driven by environmental changes such as altitude. Geographical environmental differences can affect genetic variation and evolutionary processes among species, and genomic differentiation is achieved in polyploid organisms [43]. In studies on environmental impacts on plants, altitude-an integrated reflection of vertical zonal environmental factors including temperature, humidity, latitude, and longitude-exerts a crucial influence on plant genomic variation and evolution [44]. Scholars found that the genetic diversity within populations of hexaploid *E*. *nutans* and tetraploid *Elymus breviaristatus* gradually increased from the lowest sampling site (2800 m above sea level) with rising altitude, stabilized at medium altitudes, and then decreased with further increases in altitude [45]. There was a significant altitudinal distribution pattern in the genomic size variation in *Elymus* species: populations at medium altitudes (3900–4300 m) showed greater genomic size variation than those at other altitudes, indicating that altitude shapes the evolutionary process of the genome [24]. The genetic diversity of four *Picea likiangensis* populations from the Qinghai–Tibet Plateau also showed a low-high-low pattern along the altitude gradient, with the medium-altitude population (3050 m) having higher genetic diversity than low-altitude (2900 m) and high-altitude (3200 m and 3350 m) populations [46]. Walter et al. [47] found that the increase in relatively adaptive genetic variation in *Senecio chrysanthemifolius* in new environments was closely associated with expression changes in key genes in high-altitude habitats, indicating that genetic variation can enhance population adaptive potential in new environments. A study on the genetic diversity and gene expression diversity of *Batrachium bungei* (an aquatic plant from Qinghai–Tibet Plateau) revealed that as altitude increases and environmental conditions deteriorate, the role of genetic diversity in maintaining population stability strengthens [48]. These studies support the results of this research to a certain extent. During plant polyploidization, certain subgenomes exhibited biased retention of gene families closely associated with environmental stress responses [49]. Studies have demonstrated that biased expression of the Y subgenome, which exhibited elevated transcriptional activity in response to plateau environmental stresses and thereby governed the expression of adaptive traits, played a critical role in the high-altitude adaptation of allotetraploid *Isoetes sinensis* [50]. The present study found that the St subgenome displayed substantial variation in high-altitude materials, which may be attributed to the fact that the St subgenome harbored more key adaptive genes responding to extreme cold and radiation stresses. In environments with moderate and fluctuating stress, plants-especially polyploid lineages-evolve shifted resource allocation strategies that prioritize growth competitiveness and dynamic environmental plasticity, enabling them to diverge and occupy distinct ecological niches [51]. The Y genome of middle-altitude population may undertake more regulatory functions related to growth under benign environments, maintaining the balance between growth and defense via genetic variation. Such functional division may result in divergent degrees of genomic variation at different elevations. However, further studies are needed to supplement direct genetic functional evidence through transcriptome analysis, qPCR validation, and stress physiological measurements. The present study used molecular cytogenetic methods to analyze the chromosomal structural polymorphism of *R. nutans* at different altitudes and its correlation with altitude. How environmental factors drive plant genetic evolution deserves further investigation in the future. Nevertheless, this study has several limitations. The plant materials were collected from only three altitude populations in Garzê Tibetan Autonomous Prefecture with a relatively single sampling area, restricting the generalizability of the results to the entire Western Sichuan Plateau. In addition, the limited number of altitude gradients weakened the statistical support for the correlation analysis between environmental factors and genetic characteristics. Future studies will expand the sampling range and increase altitude sampling sites to further verify and improve the research conclusions.

## 5. Conclusions

GISH and ND-FISH with two tandem repeat probes pAs1 and (AAG)_10_) elucidated the distribution of repetitive sequences on the St and Y genomes of *R*. *nutans* from the Western Sichuan Plateau. It was found that inter-population variation exceeded intra-population variation with respect to both the number of probe signals and the number of variant types. In the St genome, the number of (AAG)_10_ loci showed a moderate decrease at medium altitude and a substantial increase at high altitude along the elevational gradient. The pAs1 probe exhibited higher signal density than (AAG)_10_, with its signal abundance peaking at medium altitude, and polymorphic loci were concentrated in high-altitude populations. By contrast, medium-altitude samples harboured fewer (AAG)_10_ signals within the Y genome. Although pAs1 showed lower signal density than (AAG)_10_ in the Y genome, its maximum signal number was detected in medium-altitude populations, where polymorphic loci predominated. Low-altitude populations maintained relatively conserved hybridization patterns. Chromosomal variation was mainly observed in the Y genome at medium altitudes and in the St genome at high altitudes.

## Figures and Tables

**Table 1:** Source of *Roegneria nutans* accessions.

Accession No.	Altitude (m)	Habitat	Source
RNU0403	2939	Roadside grassy slope	Kangding City, Garzê Tibetan Autonomous Prefecture, Sichuan Province, China
RNU0408	3777	Roadside grassy slope	Kangding City, Garzê Tibetan Autonomous Prefecture, Sichuan Province, China
RNU0416	4278	Roadside grassy slope	Kangding City, Garzê Tibetan Autonomous Prefecture, Sichuan Province, China

**Table 2:** Summary of hybridization site numbers and polymorphic site counts for repetitive sequences in three *R. nutans* populations.

Population	Parameter	St Genome		Y Genome	
		(AAG)_10_	pAs1	(AAG)_10_	pAs1
R1	Average number of hybridization sites	8 b	12.20 ± 2.05 c	18.20 ± 0.84 a	6.60 ± 0.89 c
	Polymorphic sites	0	1 (5″, 1)	4 (4″, 2, 7″, 2)	0
R2	Average number of hybridization sites	5.20 ± 2.59 b	28.60 ± 2.61 a	16.80 ± 2.28 b	16.80 ± 3.27 a
	Polymorphic sites	0	2 (3″, 1, 5″, 1)	4 (3″, 1, 6″, 2, 7″, 1)	2 (3″, 1, 6″, 1)
R3	Average number of hybridization sites	16.20 ± 3.19 a	20.00 ± 1.41 b	18.20 ± 1.10 a	10.80 ± 0.84 b
	Polymorphic sites	10 (1″, 1, 2″, 1, 3″, 1, 4″, 1, 5″, 2, 6″, 4)	3 (1″, 2, 6″, 1)	2 (5″, 1, 6″, 1)	1 (3″, 1)
Total polymorphic sites		10 (1″, 1, 2″, 1, 3″, 1, 4″, 1, 5″, 2, 6″, 4)	6 (1″, 2, 3″, 1, 5″, 2, 6″, 1)	10 (3″, 1, 4″, 2, 5″, 1, 6″, 3, 7″, 3)	3 (3″, 2, 6″, 1)

Annotation: The number before the parenthesis indicates the number of hybridization signal loci, the number in the parenthesis represents the corresponding specific chromosome (Figure 2), the double prime (″) after the number denotes one pair of homologous chromosomes. For the total polymorphic loci, the number following the chromosome represents the total number of polymorphic loci possessed by that chromosome. Different letters within the same column indicate significant differences among populations (ANOVA, *p* < 0.05). Detailed individual data are provided in Table S1 in the Supplementary Materials.

**Table 3:** The chromosomal variations in *R. nutans* samples from three different altitudes were characterized using the probe combination of (AAG)_10_ and pAs1.

Accession No.	Altitude (m)	St Genome							Y Genome						
		1	2	3	4	5	6	7	1	2	3	4	5	6	7
R1-1	2939	A	A	A	A	A	A	A	A	A	A	A	A	A	A
R1-2		A	B	A	A	A	A	A	A	A	A	B	A	A	A
R1-3		A	A	A	A	A	A	A	A	A	A	C	A	A	AB
R1-4		A	B	A	A	A	A	A	A	A	A	B	B	A	A
R1-5		A	A	A	A	A	B	A	A	B	A	C	A	B	AB
No. of variant types		1	2	1	1	1	2	1	1	2	1	3	2	2	2
R2-1	3777	B	C	B	B	BC	C	B	B	C	B	D	C	CD	C
R2-2		C	C	CD	C	C	D	B	C	D	B	E	D	EF	D
R2-3		C	D	B	C	D	E	B	D	C	CD	E	C	E	DE
R2-4		C	D	B	D	C	E	B	E	D	B	E	C	G	F
R2-5		C	C	D	C	C	F	B	B	D	B	F	D	F	F
No. of variant types		2	2	3	3	3	3	1	4	2	3	3	2	5	4
R3-1	4278	DE	E	E	E	E	GH	C	C	E	E	G	E	H	G
R3-2		DE	F	E	E	EF	G	D	C	E	E	G	E	H	G
R3-3		E	E	E	FG	FG	GI	D	C	E	E	H	E	HI	H
R3-4		DE	E	EF	E	F	JK	D	C	E	E	H	E	J	I
R3-5		EF	G	E	H	F	LM	D	C	E	EF	H	F	J	I
No. of variant types		3	3	2	4	3	7	2	1	1	2	2	2	3	3
No. of total variant types		6	7	6	8	7	13	4	5	5	6	8	6	10	9
Total								51							49

Annotation: Taking the signal pattern of each numbered chromosome of individual R1-1 as the reference type, it is denoted as type A. For the chromosomes of other materials under the same genome and the same number, conduct comparison and classification according to the signal patterns: those with signal patterns consistent with the reference type are classified into the same type; when a new signal pattern different from the existing types appears, it is named sequentially as in alphabetical order, which is the variant type of that chromosome number.

## Data Availability

The original contributions presented in this study are included in the article/Supplementary Materials. Further inquiries can be directed to the corresponding author.

## Supplementary Materials

The following supporting information can be downloaded at: https://www.mdpi.com/article/10.3390/plants15182832/s1, Table S1: Hybridization site number and chromosomal distribution of repetitive sequences on individual chromosomes in three *Roegneria nutans* populations.
