# Supplementary material for: Characterization of Chromosomal Structural Polymorphism in Roegneria nutans from the Western Sichuan Plateau, China Based on Tandem Repeat Sequences
Source: Plants (Basel). 2026 Sep 16;15(18):2832. doi: 10.3390/plants15182832 (PMC13610300; doi:10.3390/plants15182832)
Supplement: Supplementary file 1 [file plants-15-02832-s001.zip › plants-4530441-supplementary.pdf]

Table S1. Hybridization site number and chromosomal distribution of repetitive sequences on individual chromosomes in three *Roegneria nutans* populations

Accession No.	St genome		Y genome	
	（AAG）10	pAs1	（AAG）10	pAs1
R1-1	8(1”,2,2”,2,3”,1,4”,1,5”,1,7”,1)	12(1”,1,2”,2,3”,2,4”,2,5”,2,6”,3)	18(1”,1,2”,2,3”,2,4”＊,4,5”,3,6”,3,7”,3)	6(1”,4,2”,1,3”,1)
R1-2	8(1”,2,2”,2,3”,1,4”,1,5”,1,7”,1)	14(1”,1,2”,4,3”,2,4”,2,5”,2,6”,3)	19(1”,1,2”,2,3”,2,4”,5,5”,3,6”,3,7”,3)	7(1”,4,2”,1,3”,1,4”,1)
R1-3	8(1”,2,2”,2,3”,1,4”,1,5”,1,7”,1)	12(1”,1,2”,2,3”,2,4”,2,5”,2,6”,3)	18(1”,1,2”,1,3”,2,4”＊,5,5”,3,6”,3,7”＊,3)	6(1”,4,2”,1,3”,1)
R1-4	8(1”,2,2”,2,3”,1,4”,1,5”,1,7”,1)	14(1”,1,2”,4,3”,2,4”,2,5”,2,6”,3)	19(1”,1,2”,2,3”,2,4”,5,5”,3,6”,3,7”,3)	8(1”,4,2”,1,3”,1,4”,1,5”,1)
R1-5	8(1”,2,2”,2,3”,1,4”,1,5”,1,7”,1)	9(1”,1,2”,2,3”,2,4”,2,5”＊,2)	17(1”,1,2”,1,3”,2,4”,5,5”,3,6”,2,7”＊,3)	6(1”,4,2”,1,3”,1)
R2-1	3(1”,1,3”,2)	24(1”,2,2”,4,3”,6,4”,3,5”＊,4,6”,3,7”,2)	14(1”,2,3”,3,4”,2,5”,2,6”＊,3,7”,2)	14(1”,4,2”,1,3”,1,4”,2,5”,2,6”,2,7”,2)
R2-2	3(1”,1,3”,1,6”,1)	30(1”,4,2”,4,3”＊,8,4”,5,5”,4,6”,3,7”,2)	18(1”,3,2”,1,3”,3,4”,2,5”,3,6”,3,7”,3)	18(1”,4,2”,1,3”,1,4”,3,5”,2,6”＊,3,7”,4)
R2-3	8(1”,1,2”,2,3”,1,5”,1,6”,2)	30(1”,4,2”,4,3”,6,4”,5,5”,5,6”,4,7”,2)	20(1”,3,3”＊,4,4”,2,5”,2,6”,3,7”＊,6)	22(1”,5,2”,1,3”＊,3,4”,3,5”,2,6”,4,7”,4)
R2-4	8(1”,1,2”,2,3”,2,4”,1,6”,2)	29(1”,4,2”,4,3”,6,4”,4,5”,5,6”,4,7”,2)	16(1”,2,2”,1,3”,3,4”,2,5”,2,6”＊,3,7”,3)	15(1”,5,2”,1,3”,1,4”,3,5”,2,6”,2,7”,1)
R2-5	4(1”,1,3”,1,7”,2)	30(1”,4,2”,4,3”,8,4”,5,5”,4,6”,3,7”,2)	16(1”,2,2”,1,3”,3,4”,2,5”,3,6”,2,7”,3)	15(1”,4,2”,1,3”,1,4”,2,5”,2,6”,3,7”,2)
R3-1	18(1”,4,2”＊,3,3”,2,4”,2,5”,2,6”＊,4,7”,1)	18(1”＊,3,2”,4,3”,2,4”,2,5”,2,6”,5)	19(1”,1,2”,1,3”,2,4”,3,5”,4,6”,4,7”,4)	10(1”,3,2”,2,3”,2,4”,1,5”,1,6”,1)
R3-2	17(1”,4,2”,2,3”,2,4”,2,5”＊,2,6”,4,7”,1)	19(1”＊,3,2”,4,3”,2,4”,2,5”,2,6”,5,7”,1)	19(1”,1,2”,1,3”,2,4”,3,5”,4,6”,4,7”,4)	10(1”,3,2”,2,3”,2,4”,1,5”,1,6”,1)
R3-3	20(1”,4,2”,3,3”,2,4” ＊ ,4,5”＊,3,6”＊,3,7”,1)	21(1”,4,2”,4,3”,2,4”,2,5”,2,6”＊,6,7”,1)	19(1”,1,2”,1,3”,2,4”,3,5”,4,6”＊,4,7”,4)	12(1”,3,2”,2,3”,2,4”,2,5”,1,,6”,1,7”,1)
R3-4	14(1”,3,2”,2,3” ＊ ,2,4”,2,6”＊,4,7”,1)	21(1”,3,2”,4,3”,2,4”,2,5”,2,6”,7,7”,1)	17(1”,1,2”,1,3”,2,4”,3,5”＊,4,6”,3,7”,3)	11(1”,3,2”,2,3”,2,4”,2,5”,1,6”,1)
R3-5	12(1” ＊ ,3,2”,1,3”,2,4”,1,6”＊,4,7”,1)	21(1”,3,2”,4,3”,2,4”,3,5”,2,6”,6,7”,1)	17(1”,1,2”,1,3”,2,4”,3,5”,4,6”,3,7”,3)	11(1”,3,2”,2,3”＊,2,4”,2,5”,1,6”,1)

Annotation: The number before the parenthesis indicates the number of hybridization signal loci, the number in the parenthesis represents the corresponding specific chromosome (Figure 2), the double prime (″) after the number denotes one pair of homologous chromosomes, and the asterisk (*) indicates that chromosomes exhibit polymorphism between two individuals of the same population.
